# Predicting the rate of inbreeding in populations undergoing four-path selection on genomically enhanced breeding values

**DOI:** 10.5713/ab.21.0350

**Published:** 2022-01-03

**Authors:** Kenji Togashi, Kazunori Adachi, Kazuhito Kurogi, Takanori Yasumori, Toshio Watanabe, Shohei Toda, Satoshi Matsubara, Kiyohide Hirohama, Tsutomu Takahashi, Shoichi Matsuo

**Affiliations:** 1 Maebashi Institute of Animal Science, Livestock Improvement Association of Japan, Maebashi, Gunma, 371-0121, Japan; 2 Livestock Improvement Association of Japan, Koto-ku, Tokyo 135-0041, Japan; 3 Maebashi Bull Center, Livestock Improvement Association of Japan, Maebashi, Gunma, 371-0121, Japan; 4 Tokachi Bull Center, Livestock Improvement Association of Japan, Nakagawagun, Hokkaido, 089-0625, Japan

**Keywords:** Equilibrium Genetic Variances, Four-path Selection, Long-time Genetic Contribution, Rates of Inbreeding, Selective Advantage

## Abstract

**Objective:**

A formula is needed that is practical for current livestock breeding methods and that predicts the approximate rate of inbreeding (ΔF) in populations where selection is performed according to four-path programs (sires to breed sons, sires to breed daughters, dams to breed sons, and dams to breed daughters). The formula widely used to predict inbreeding neglects selection, we need to develop a new formula that can be applied with or without selection.

**Methods:**

The core of the prediction is to incorporate the long-tern genetic influence of the selected parents in four-selection paths executed as sires to breed sons, sires to breed daughters, dams to breed sons, and dams to breed daughters. The rate of inbreeding was computed as the magnitude that is proportional to the sum of squared long-term genetic contributions of the parents of four-selection paths to the selected offspring.

**Results:**

We developed a formula to predict the rate of inbreeding in populations undergoing four-path selection on genomically enhanced breeding values and with discrete generations. The new formula can be applied with or without selection. Neglecting the effects of selection led to underestimation of the rate of inbreeding by 40% to 45%.

**Conclusion:**

The formula we developed here would be highly useful as a practical method for predicting the approximate rate of inbreeding (ΔF) in populations where selection is performed according to four-path programs.

## INTRODUCTION

Deterministic predictions of response to multi-trait genomic selection in a single generation in a population with four-path programs, was developed [1,2]. That is, the selection paths in four-path programs are sires to breed sires (SS), sires to breed dams (SD), dams to breed sires (DS), and dams to breed dams (DD). However, when creating formulas for calculating the asymptotic response to index or single-trait selection in four-path selection programs rather than in a single generation, the initial genetic response in generation 0 overestimated the asymptotic response due to the decrease in equilibrium genetic variance from generation 0 onwards [3]. Consequently, to safeguard the genetic variation of the population over the long term, the rate of inbreeding needs to be restricted to an acceptable level. Therefore, one needs to know the expected rate of inbreeding as well as the equilibrium genetic response before choosing a breeding scheme.

A population with discrete generations under mass selection in a four-path selection program is modeled to predict the rate of inbreeding in the long term. When sires in the SS path are used with constant selection intensity and in equal number throughout the usage period of several years, every SS sire belongs to a single or exclusive category. Similarly, SD, DS, and DD parents each belong to a single or exclusive category when they are used with constant selection intensity and in equal numbers over several years. Consequently generations can be regarded as discrete rather than overlapping. A formula is needed that is practical for current livestock breeding methods and that predicts the approximate rate of inbreeding (ΔF) in populations where selection is performed according to four-path programs.

The rate of inbreeding is proportional to the sum of squared long-term genetic contributions [4]. General predictions of expected genetic contributions were developed by Woolliams et al [5] by using equilibrium genetic variances instead of second-generation genetic variances. Methods were developed by Bijma and Woolliams [6] to predict rates of inbreeding in populations selected on breeding values according to best linear unbiased prediction (BLUP) [7]. A formula was developed for predicting the rate of inbreeding in four-selection path programs [8]; however, this formula ignored the effect of selection. The purpose of the current study was to develop a formula for predicting the rate of inbreeding in four-path selection programs that incorporated the effect of selection and was practical for use under real-life conditions of cattle breeding.

## MATERIALS AND METHODS

### Prediction of expected long-term genetic contributions

Our prediction method is based on the concept of long-term genetic contributions. The long-term genetic contribution of individual i (*r_i_*) in generation t_1_ is defined as the proportion of genes from individual i that are present in individuals in generation t_2_ deriving by descent from individual i, where (t_2_–t_1_) →∞ [5]. That is, after several generations, the genetic contributions of ancestors stabilize and become equal for all descendants, i.e., the ultimate proportional contribution of an ancestor to its descendants is reached.

Selection is performed in four categories of selection path (SS, SD, DS, and DD). Rates of inbreeding can be expressed in terms of the expected contributions of these categories [6, 9–11]:


$$\text{E }(\Delta \text{F})=\frac{1}{2}1^{\prime }N\text{E }(u^{2})+\frac{1}{8}1^{\prime }N\delta ,$$

where **1′ = (1 1 1 1), N** is a 4×4 diagonal matrix containing the number of selected parents for element (i, i) as N_i,i_, N_1,1_ is the number of sires in SS and is referred to as *N_SS_*, N_2,2_ is the number of sires in SD and is referred to as *N_SD_*, N_3,3_ is the number of dams in DS and is referred to as *N_DS_*, and N_4,4_ is the number of dams in DD and is referred to as *N_DD_*. In addition, $u^{2}=(u_{i,SS}^{2}\;u_{i,SD}^{2}\;u_{i,DS}^{2}\;u_{i,DD}^{2})$, where *u_i,SS_* is the expected lifetime long-term genetic contribution of individual i in category SS conditional on its selective advantage (which in mass selection is the genomically enhanced breeding value [GEBV]), and *u_i,SD_*, *u_i,DS_*, and *u_i,DD_* are the expected lifetime long-term genetic contributions of individual i in categories SD, DS, and DD, respectively. Furthermore, **δ** = (δ*_SS_* δ*_SD_* δ*_DS_* δ*_DD_*), where δ*_SS_* is the correction factor for deviations of the variance of family size from independent Poisson variances in the selected offspring from sires in SS; δ*_SD_*, δ*_DS_*, and δ*_DD_* are corrections for deviations of the variance of the family size from independent Poisson variances in the selected offspring from parents in SD, DS, and DD, respectively.

The selective advantage of the ith sire in SS (*S_i,SS_*) and in SD (*S_i,SD_*) in the linear model is:


$$\begin{array}{l} s_{i,SS}=A_{i,SS}+{\bar{A}}_{i,DS}-({\bar{A}}_{SS}+{\bar{A}}_{DS})\;\text{and}\\ s_{i,SD}=A_{i,SD}+{\bar{A}}_{i,DD}-({\bar{A}}_{SD}+{\bar{A}}_{DD}),\text{respectively},\; \end{array}$$

where *A_i,SS_* is the breeding value of sire i in SS or SD, *Ā_i,DS and DD_* is the average breeding value of dams mated to the ith sire in SS and SD, respectively; the dams mated to the ith sire in SS belong to the DS category, and the dams mated to the ith sire in SD belong to the DD category; and *Ā_i,SS_*, *Ā_i,SD_*, *Ā_i,DS_*, and *Ā_i,DD_*, are the average breeding values of the individuals in the SS, SD, DS, and DD categories.

The selective advantage of the ith dam in DS (*S_i,DS_*) and in DD (*S_i,DD_*) in the linear model is:


$$\begin{array}{l} s_{i,DS}=A_{i,DS}+A_{i,SS}-({\bar{A}}_{DS}+{\bar{A}}_{SS})\;\text{and}\\ s_{i,DD}=A_{i,DD}+A_{i,SD}-({\bar{A}}_{DD}+{\bar{A}}_{SD}),\text{respectively},\; \end{array}$$

where *A_i,DS and DD_* is the breeding value of dam i in DS and DD, respectively; *A_i,SS and SD_* is the breeding value of a sire mated to the ith dam in DS and DD, respectively; the sires mated to the ith dam in DS belong to the SS category; and the sires mated to the ith dam in DD belong to the SD category.

Expected contributions (*u_i,SS,SD,DS,or DD_*) are predicted by linear regression on the selective advantage. That is,


$$u_{i,x}=\text{E }(r_{i}|s_{i,x})=\alpha_{x}+\beta_{x}s_{i,\;x},x=\text{SS},\text{SD},\text{DS},\text{or DD},$$

where *α_x_* is the expected contribution of an average parent in *x*, and *β_x_* is the regression coefficient of the contribution of i on its selective advantage (*S_i,x_*). In addition, *α_x_* can be obtained according to Woolliams et al [9]:


$$N\alpha =G^{\prime }N\alpha ,$$

where **G** is a 4×4 matrix representing the parental origin of genes of selected offspring in the order of SS, SD, DS, and DD category, i.e., representing rows offspring and columns parental categories. That is,


$$G_{4\times 4}=\left[\begin{array}{ccccc} & SS & SD & DS & DD\\ SS & 0.5 & 0 & 0.5 & 0\\ SD & 0.5 & 0 & 0.5 & 0\\ DS & 0 & 0.5 & 0 & 0.5\\ DD & 0 & 0.5 & 0 & 0.5 \end{array}\right].$$

However,


$$\alpha^{\prime }N=\alpha^{\prime }\mathrm{NG},$$

where **α′N** is the left eigenvector of **G** with eigenvalue 1; the left eigenvector is obtained according to Bijma and Woolliams [11] and is equal to (0.25 0.25 0.25 0.25).


$$\text{Therefore }\alpha^{\prime }=(\frac{1}{4N_{SS}}\frac{1}{4N_{SD}}\frac{1}{4N_{DS}}\frac{1}{4N_{DD}}).$$

Solutions for *β_x_* are obtained according to Woolliams et al [9]:


(1) $$\begin{array}{l} \left[\begin{array}{c} N_{SS}\beta_{SS}\\ N_{SD}\beta_{SD}\\ N_{DS}\beta_{DS}\\ N_{DD}\beta_{DD} \end{array}\right]={\left(I_{4}-\frac{1}{2}\Pi^{\prime }\right)}^{-1}\times \frac{1}{2}\Lambda^{\prime }\times \left[\begin{array}{c} N_{SS}\alpha_{SS}\\ N_{SD}\alpha_{SD}\\ N_{DS}\alpha_{DS}\\ N_{DD}\alpha_{DD} \end{array}\right]\\ ={\left(I_{4}-\frac{1}{2}\Pi^{\prime }\right)}^{-1}\times \frac{1}{2}\Lambda^{\prime }\times \left[\begin{array}{c} \frac{1}{4}\\ \frac{1}{4}\\ \frac{1}{4}\\ \frac{1}{4} \end{array}\right], \end{array}$$

note that the right hand side of (1) is unaffected by the number of parents, so that *β_x_* is inversely proportional to the number of parents (that is, $\frac{1}{N_{SS}},\frac{1}{N_{SD}},\frac{1}{N_{DS}}$ and $\frac{1}{N_{DD}}$), where, **I**_4_ is a 4×4 identity matrix, **Π** is a 4×4 matrix of regression coefficients with *π_xy_* being the regression coefficient of *S_i,x_* of a selected offspring i of category *x* (SS, SD, DS, DD) on *s_j,y_* of its parent j of category *y* (SS, SD, DS, DD). For example, *π_SD,SS_* is the regression coefficient of *S_i,SD_* of a selected offspring i of SD on *S_j,SS_* of its parent j of SS. Given that SS is the sires to breed sons category, we have non-zero elements, *π_SS,SS_* and *π_SD,SS_*, in **Π** as elements (1,1) and (2,1), respectively. In the same way, since SD is the sires to breed daughters category, we have non-zero elements, *π_DS,SD_* and *π_DD,SD_*, in **Π** as elements (3,2) and (4,2), respectively. Because DS is the dams to breed sons category, we have non-zero elements, *π_SS,DS_* and *π_SD,DS_*, in **Π** as elements (1,3) and (2,3), respectively. And given that DD is the dams to breed daughters category, we have non-zero elements, *π_DS,DD_* and *π_DD,DD_*, in **Π** as elements (3,4) and (4,4), respectively.

In addition, **Λ** is a 4×4 matrix of regression coefficients, with *λ_xy_* being the regression coefficient of the number of selected offspring of category *x* on *S_j,y_* of its parent j of category *y*. In the same way as **Π**, we have non-zero elements, *λ_SS,SS_* and *λ_SD,SS_*, *λ_DS,SD_* and *λ_DD,SD_*, *λ_SS,DS_* and *λ_SD,DS_*, and *λ_DS,DD_* and *λ_DD,DD_* in **Λ** as elements (1,1) and (2,1), (3,2) and (4,2), (1,3) and (2,3), and (3,4) and (4,4), respectively. Consequently,


$$\begin{array}{l} \Pi_{4\times 4}=\left[\begin{array}{ccccc} & SS & SD & DS & DD\\ SS & \pi_{SS,SS} & 0 & \pi_{SS,DS} & 0\\ SD & \pi_{SD,SS} & 0 & \pi_{SD,DS} & 0\\ DS & 0 & \pi_{DS,SD} & 0 & \pi_{DS,DD}\\ DD & 0 & \pi_{DD,SD} & 0 & \pi_{DD,DD} \end{array}\right],\text{and}\\ \Lambda_{4\times 4}=\left[\begin{array}{ccccc} & SS & SD & DS & DD\\ SS & \lambda_{SS,SS} & 0 & \lambda_{SS,DS} & 0\\ SD & \lambda_{SD,SS} & 0 & \lambda_{SD,DS} & 0\\ DS & 0 & \lambda_{DS,SD} & 0 & \lambda_{DS,DD}\\ DD & 0 & \lambda_{DD,SD} & 0 & \lambda_{DD,DD} \end{array}\right], \end{array}$$

representing rows as offspring and columns as parental categories.

In our current study, elements in matrices **Π** and **λ** were calculated from Woolliams et al [9] and Bijma and Woolliams [11], as outlined in Appendices A and B.

The sires in the SS category are included among the sires in SD category. That is, the sires in the SS category are selected not only to breed sons but as sires in the SD category to breed daughters. Similarly the dams in the DS category are included among the dams in the DD category. The dams in the DS category are selected not only to breed sons but as dams in the DD category to breed daughters. Therefore, after applying the procedure of Bijma and Woolliams [6], the number of sires in SD is larger than that of sires in SS, and the number of dams in DD is larger than that of dams in DS. Therefore, $\text{E }(\Delta \text{F})=\frac{1}{2}(1^{\prime }N_{0}U_{0}1)$, where


$$\begin{array}{l} N_{0}=\left[\begin{array}{cccc} N_{SD} & 0 & 0 & 0\\ 0 & N_{SS} & 0 & 0\\ 0 & 0 & N_{DD} & 0\\ 0 & 0 & 0 & N_{DS} \end{array}\right],\\ U_{0}=\left[\begin{array}{cccc} E({\text{u}}_{i,SD}^{2}) & 0 & 0 & 0\\ 2E(u_{i,SD}u_{i,SS}) & \text{E }({\text{u}}_{i,SS}^{2}) & 0 & 0\\ 0 & 0 & E({\text{u}}_{i,DD}^{2}) & 0\\ 0 & 0 & 2E(u_{i,DD}u_{i,DS}) & E({\text{u}}_{i,DS}^{2}) \end{array}\right], \end{array}$$

*E* denotes the expectation with respect to the selective advantage,


$$\begin{array}{l} E\;\left({\text{u}}_{i,SS}^{2}\right)=\alpha_{SS}^{2}+\beta_{SS}^{2}\;\sigma_{SS}^{2},\;E({\text{u}}_{i,SD}^{2})=\alpha_{SD}^{2}+\beta_{SD}^{2}\;\sigma_{SD}^{2},\\ E\;\left({\text{u}}_{i,DS}^{2}\right)=\alpha_{DS}^{2}+\beta_{DS}^{2}\;\sigma_{DS}^{2},\;E({\text{u}}_{i,DD}^{2})=\alpha_{DD}^{2}+\beta_{DD}^{2}\;\sigma_{DD}^{2},\\ \sigma_{SS}^{2}=\sigma_{A,m}^{2}\;\left(1-k_{SS}r_{\hat{A},m}^{2}\right)\;\left(1-\frac{1}{N_{SS}}\right)+\frac{\sigma_{A,f}^{2}}{\frac{N_{DS}}{N_{SS}}}\left(1-k_{DS}r_{\hat{A},f}^{2}\right)\;\left(1-\frac{1}{N_{SS}}\right),\\ \sigma_{SD}^{2}=\sigma_{A,m}^{2}\;\left(1-k_{SD}r_{\hat{A},m}^{2}\right)\;\left(1-\frac{1}{N_{SD}}\right)+\frac{\sigma_{A,f}^{2}}{\frac{N_{DD}}{N_{SD}}}\left(1-k_{DD}r_{\hat{A},f}^{2}\right)\;\left(1-\frac{1}{N_{SD}}\right),\\ \sigma_{DS}^{2}=\sigma_{A,f}^{2}\;\left(1-k_{DS}r_{\hat{A},f}^{2}\right)\;\left(1-\frac{1}{N_{DS}}\right)+\sigma_{A,m}^{2}\;\left(1-k_{SS}r_{\hat{A},m}^{2}\right)\;\left(1-\frac{1}{N_{SS}}\right),\\ \sigma_{DD}^{2}=\sigma_{A,f}^{2}\;\left(1-k_{DD}r_{\hat{A},f}^{2}\right)\;\left(1-\frac{1}{N_{DD}}\right)+\sigma_{A,m}^{2}\;\left(1-k_{SD}r_{\hat{A},m}^{2}\right)\;\left(1-\frac{1}{N_{SD}}\right),\\ E(u_{i,SD}u_{i,SS})=\alpha_{SD}\alpha_{SS}+\beta_{SD}\beta_{SS}\sigma_{A,m}^{2}\;\left(1-k_{SS}r_{{\hat{A}}_{m}}^{2}\right)\;\left(1-\frac{1}{N_{SS}}\right)+\alpha_{SS}\beta_{SD}E({\bar{\text{A}}}_{\text{SS}}-{\bar{\text{A}}}_{\text{SD}}),\\ E({\bar{\text{A}}}_{\text{SS}}-{\bar{\text{A}}}_{\text{SD}})=(i_{SS}-i_{SD})\sigma_{A,m},\\ E(u_{i,DS}u_{i,DD})=\alpha_{DS}\alpha_{DD}+\beta_{DS}\beta_{DD}\sigma_{A,f}^{2}\;\left(1-k_{DS}r_{{\hat{A}}_{f}}^{2}\right)\;\left(1-\frac{1}{N_{DS}}\right)+\alpha_{DS}\beta_{DD}E({\bar{A}}_{DS}-{\bar{A}}_{DD}), \end{array}$$

and *E*(*Ā_DS_* – *Ā_DD_*) = (*i_DS_* – *i_DD_*)*σ_A,f_*,

note that variance of selective advantage ( $\sigma_{SS}^{2},\sigma_{SD}^{2},\sigma_{DS}^{2}$, and $\sigma_{DD}^{2}$) is not affected greatly by the number of parents (*N_SS_*, *N_SD_*, *N_DS_*, and *N_DD_*), since the term of $\left(1-\frac{1}{N_{x}}\right)$ is adjustment for finite population size, where $\sigma_{A,m}^{2}$ and $\sigma_{A,f}^{2}$ are the equilibrium genetic variance in the male and female populations, respectively; $r_{\hat{A},m}^{2}$ and $r_{{\hat{A}}_{f}}^{2}$ are the equilibrium reliability of GEBV in the male and female populations, respectively; and *k_SS_*, *k_SD_*, *k_DS_*, and *k_DD_* are variance reduction coefficients for offspring selection in SS, SD, DS, and DD, respectively. Note that covariances of mates between SS and SD and between DS and DD are zero, because of random mating. General predictions of expected genetic contributions was developed using equilibrium genetic variances instead of second generation genetic variances [9]. Therefore, variances thereafter refer to those in equilibrium.

The accounting percentage derived from SS, SD, DS, and DD for the rate of inbreeding (ΔF) is obtained,


$$\begin{array}{l} {}[\frac{1}{2}N_{SS}\times \left(E(u_{i,SS}^{2}\right)+E(u_{i,SD}u_{i,SS}))]/E(\Delta F),\\ {}[\frac{1}{2}N_{SD}\times \left(E(u_{i,SD}^{2}\right)+\frac{1}{2}N_{SS}E(u_{i,SD}u_{i,SS})]/E(\Delta F),\\ {}[\frac{1}{2}N_{DS}\times \left(E(u_{i,DS}^{2}\right)+E(u_{i,DS}u_{i,DD}))]/E(\Delta F),\text{and}\\ {}[\frac{1}{2}N_{DD}\times E(u_{i,DD}^{2})+\frac{1}{2}N_{DS}E(u_{i,DS}u_{i,DD})]/E(\Delta F),\text{respectively.} \end{array}$$

When the effect of selection on inbreeding is ignored, i.e., β = 0, $\text{E }(\Delta \text{F})=\frac{1}{2}(1^{\prime }N_{0}U_{0}1)=\frac{1}{32}(\frac{1}{N_{SS}}+\frac{3}{N_{SD}}+\frac{1}{N_{DS}}+\frac{3}{N_{DD}})$.

This result is in agreement with the formula from Gowe et al [8], which likewise neglects the effects of selection on ΔF.

### Correction of E (ΔF) from Poisson variances

The correction for deviations of the variance of the family size from independent Poisson variances in the selected offspring from SS, SD, DS, and DD parents, i.e., δ*_SS_*, δ*_SD_*, δ*_DS_*, and δ*_DD_*, can be approximated by Woolliams and Bijma [10].

According to Woolliams and Bijma [10],


$$\begin{array}{l} \delta_{SS}=\text{E }(u_{\mathrm{SS}}^{*T}\Delta V_{\mathrm{SS}}u_{\mathrm{SS}}^{*}),\delta_{\text{SD}}=\text{E }(u_{\mathrm{SD}}^{*T}\Delta V_{\mathrm{SD}}u_{\mathrm{SD}}^{*}),\\ \delta_{\text{DS}}=\text{E }(u_{\mathrm{DS}}^{*T}\Delta V_{\mathrm{DS}}u_{\mathrm{DS}}^{*}),\text{and }\delta_{\text{DD}}=\text{E }(u_{\mathrm{DD}}^{*T}\Delta V_{\mathrm{DD}}u_{\mathrm{DD}}^{*}), \end{array}$$

Where


$$\begin{array}{l} u_{\mathrm{SS}}^{*}=\left[\begin{array}{c} \alpha_{SS}+\beta_{SS}\pi_{SS,SS}s_{SS}\\ \alpha_{SD}+\beta_{SD}\pi_{SD,SS}s_{SS}\\ 0\\ 0 \end{array}\right],u_{\mathrm{SD}}^{*}=\left[\begin{array}{c} 0\\ 0\\ \alpha_{DS}+\beta_{DS}\pi_{DS,SD}s_{SD}\\ \alpha_{DD}+\beta_{DD}\pi_{DD,SD}s_{SD} \end{array}\right],\\ u_{\mathrm{DS}}^{*}=\left[\begin{array}{c} \alpha_{SS}+\beta_{SS}\pi_{SS,DS}s_{DS}\\ \alpha_{SD}+\beta_{SD}\pi_{SD,DS}s_{DS}\\ 0\\ 0 \end{array}\right],\\ \text{and }u_{\mathrm{DD}}^{*}=\left[\begin{array}{c} 0\\ 0\\ \alpha_{DS}+\beta_{DS}\pi_{DS,DD}s_{DD}\\ \alpha_{DD}+\beta_{DD}\pi_{DD,DD}s_{DD} \end{array}\right]; \end{array}$$

**Δ*V****_SS_***, Δ*V****_SD_***, Δ*V****_DS_*, and **Δ*V****_DD_*, are 4×4 matrices which are variances of selected family size deviated from Poisson variance by applying binomial distribution to the family size from the parents of SS, SD, DS, and DD, respectively, and *s_x_* is the selective advantage of parents in category *x* (SS, SD, DS, DD). Elements of **Δ*V****_SS,SD,DS,or DD_* are shown in Appendix C.

### Example applications of the formula

To demonstrate the application of our formula, we assumed only two quantitative traits: trait 1 was assumed to be moderately heritable, with *h*^2^ = 0.3, whereas trait 2 was assumed to have low heritability, with *h*^2^ = 0.1. These traits are selected as single traits expressed as GEBV. Furthermore, we assumed an aggregate genotype as a linear combination of genetic values, each weighted by the relative economic weights, which was expressed as *a*_1_*g*_1_ + *a*_2_*g*_2_, where *g*_1_ is the true genetic value for trait i, *a_i_* is the relative economic weight for trait i, and the genetic correlation between traits 1 and 2 was assumed as 0.4. Index selection was performed to select *a*_1_*g*_1_ + *a*_2_*g*_2_, that is, breeding goal (*H*), under the assumption that the relative economic weight between traits 1 and 2 is 1:1. Breeding value (*A*) was defined as described earlier in the Methods; for example, the breeding value of sire i in SS was defined as *A_i,SS_*. Similarly, the breeding goal value (*H*) of sire i in SS can be expressed as *H_i,SS_*; note that the formula that we developed in Methods can be applied not only to breeding value (*A*) but also to breeding goal value (*H*).

In our example, we assumed the reliabilities of the GEBVs for traits 1 and 2 to be 0.5721 and 0.4836, respectively [3]. Index selection (*I*) was performed as *I* = *a*_1_ *GEBV*_1_ + *a*_1_ *GEBV*_2_, because GEBVs are assumed to be derived from multiple-trait BLUP (MT BLUP) genetic evaluation methods in the current study (as done for single-step genomic BLUP [13]). We calculated equilibrium genetic variances and reliabilities based on Togashi et al [3]. The initial (generation 0) and equilibrium genetic variances and reliabilities for single-trait selection (*h*^2^ = 0.3 or *h*^2^ = 0.1) and index selection are shown in Table 1. Rates of inbreeding were calculated based on equilibrium genetic variances and reliabilities, because regression coefficients of the number or breeding value of selected offspring on the breeding value of the parent are equal for the parental and offspring generations under equilibrium genetic variances and reliabilities.

We considered two scenarios for the selection percentages for SS, SD, DS, and DD—5%-12.5%–1%-70% and 1%-5%–1%-70%—and three scenarios for the numbers of selected parents of SS, SD, DS, and DD—namely 20-50–100-7,000, 40-100–200-14,000, and 60-150–300-21,000. Therefore, we considered six scenarios (two scenarios of selection percentage and three scenarios of the number of parents in SS, SD, DS, DD) in total. Note that the two scenarios for selection percentage for SS, SD, DS, and DD differ only in the selection percentage along the SS and SD selection paths, because under actual breeding conditions, selection intensity can be adjusted more easily in male selection paths (SS and SD) than in female selection paths (DS and DD). The numbers of male and female offspring from a dam of DS, i.e., *fmds* and *ffds*, were set at 4. The number of female offspring from a dam of DD, i.e., *ffdd*, was set at 1.4. These numbers are derived from the years of usage of a dam and the reproduction method (ovum collection, *in vitro* fertilization, or embryo transfer). When DS and DD parents are used with constant selection intensity and in equal numbers over several years, they belong to a single or exclusive category. The numbers are used to compute the deviation of the variance of the family size from Poisson variance.

## RESULTS AND DISCUSSION

### Rates of inbreeding

The rates of inbreeding without correction for deviation from Poisson variances (that is, the rates of inbreeding with Poisson family size) are shown in Table 2. Because the rates from Gowe et al [8] do not account for selection, ΔF is the same between two selection percentages in SS, SD, DS, and DD, i.e., 1%-5%–1%-70% and 5%-12.5%–1%-70%. In contrast, ΔF derived from the method developed in the current study increased with the increase in selection intensity. When we applied our formula, ΔF was lower when selection was ignored than when it was included, suggesting that ΔF was underestimated when selection was ignored. The ratio of ΔF when selection was ignored to that when it was included was about 0.61 under the selection percentages of 5%-12.5%–1%-70% for the SS, SD, DS, and DD selection paths, whereas the ΔF ratio was 0.53 to 0.56 under the selection percentage condition of 1%-5%–1%-70%. That is, calculation according to Gowe et al [8] underestimated ΔF by approximately 40% and 45% under selection percentages of 5%-12.5%–1%-70% and 1%-5%–1%-70% for the SS, SD, DS, and DD selection paths, respectively. In contrast, the rates of inbreeding under selection estimated by using our formula were 63% to 87% greater than those calculated according to the current working formula, which does not consider selection [8]. The ratio of ΔF for 5%-12.5%–1%-70% to that for 1%-5%–1%-70% was 0.88 to 0.89, resulting in an approximately 12% decrease in ΔF due to increasing the selection percentage or decreasing the selection intensity for SS and SD for all three scenarios compared in the numbers of parents in SS, SD, DS, and DD (20-50–100-7,000, 40-100–200-14,000, and 60-150–300-21,000). In contrast, the decrease in ΔF due to the increase in the number of parents was proportional to the numbers. The ΔF under the number of parents in SS, SD, DS, and DD (40-100–200-14,000 and 60-150–300-21,000) was approximately half and one third of the ΔF under the number of parents (20-50–100-7,000), respectively, for all two scenarios compared in the selection percentage of parents in SS, SD, DS, and DD (5%-12.5%–1%-70% and 1%-5%–1%-70%). Consequently, the decrease in the rate of inbreeding likely would be greater with an increase in the number of parents than with a decrease in selection intensity; however, we need to perform more trials at different selection intensities to confirm this association.

In general, both genetic gain and ΔF increase with an in crease in selection intensity. However, because the number of parents has a greater effect on inbreeding than does selection intensity, increasing the number of parents is one option for offsetting the increase in ΔF due to an increase in selection intensity.

The rate of inbreeding was slightly lower in single-trait se lection with a low heritable trait (*h*^2^ = 0.1) than the other selection methods (i.e., single-trait selection with a trait (*h*^2^ = 0.3) and index selection [Table 2]). However, the difference was not so remarkable. Consequently, we consider the major factors in the rate of inbreeding to be the number of parents and the selection intensity in each of the four selection paths.

Values for effective population size expressed as $N_{E}=\frac{1}{2\Delta F}$ are shown in Table 3. Using a method that ignores selection [8] overestimated the effective population size due to ΔF compared with that computed by using our formula, which accounts for selection. The overestimation was greater when the selection percentage in SS, SD, DS, and DD was 1%-5%–1%-70% than when it was 5%-12.5%–1%-70%. The ratio of NE for the 5%-12.5%–1%-70% condition to that for 1%-5%–1%-70% became greater as the numbers of parents in SS, SD, DS, and DD increased from 20-50–100-7,000 to 40-100–200-14,000 and then to 60-150–300-21,000. This pattern is consistent with the suggestion that increasing the number of parents is one option for offsetting an increase in ΔF due to an increase in selection intensity (Table 2). That is, decreasing ΔF is equivalent to increasing the effective population size.

The expectation of the square of long-term contribution of an individual (that is, $E\;\left(u_{i,SS}^{2}\right),\;E\;\left(u_{i,SD}^{2}\right),\;E\;\left(u_{i,DS}^{2}\right)$, and $E\;\left(u_{i,DD}^{2}\right)$) in SS, SD, DS, and DD are shown in Table 4. The expectation of the square of long-term contribution of an individual was the greatest in SS of all the four selection paths (SS, SD, DS, and DD), since selection intensity is the highest and the number of parents is the smallest of all the four selection paths. On the contrary, the square of long-term contribution of an individual was the smallest in DD of all the four selection paths, since selection intensity in DD is the lowest and the number of parents is the largest of all the four selection paths. The square of long-term contribution of an individual in SD was greater than that in DS, mainly because the number of parents in SD is smaller than those in DS. With the increase in selection intensity or decrease in selection percentage in the four selection paths (SS-SD-DS-DD), i.e., from 5%-12.5%–1%-70% to 1%-5%–1%-70%, the increase in the square of long-term contribution of individuals in SS and DS was greater than that in SD and DD, because the selective advantage of an individual in DS was the sum of its breeding value and the breeding value of its mate in SS category with the greatest long-term contribution of all the four selection paths. The increase in the number of parents decreased the square of long-term contribution of an individual in SS, SD, DS, and DD, because the expected contribution of an average parent (*α*) in each of the four selection paths decreased with the increase in the number of parents. The square of long-term contribution of an individual was slightly lower in single-trait selection with a low heritable trait (*h*^2^ = 0.1) than the other selection methods (i.e., single-trait selection with a trait (*h*^2^ = 0.3) and index selection). However, the difference was not so remarkable in all selection methods (single-trait selection with a trait (*h*^2^ = 0.1 or 0.3) and index selection), which was consistent with the trend that the rate of inbreeding was almost the same in all selection methods (Table 2).

The accounting percentage derived from SS, SD, DS, and DD for the rate of inbreeding (ΔF) when the numbers of parents in SS, SD, DS, and DD are 40-100–200-14,000 is shown in Table 5. The accounting percentage in SS was the greatest of all the four selection paths for all two scenarios compared in the selection percentage in SS, SD, DS, and DD (that is, 5%-12.5%–1%-70% and 1%-5%–1%-70%), because the expectation of the square of lifetime long-term contribution of an individual was the greatest in SS of all the four selection paths (Table 4). The sum of accounting percentage in SS and SD was approximately 90% for ΔF, because the number of male parents in SS and SD was smaller than that of female parents in DS and DD and selection intensity in male parents is generally higher than that in female parents. In addition, the accounting percentage in each of the four selection paths when the numbers of parents in SS, SD, DS, and DD were 40-100–200-14,000 (Table 5) was approximately the same as the other scenario when the numbers of parents in SS, SD, DS, and DD were 20-50–100-7,000 or 60-150–300-21,000, although the accounting percentage in SS, SD, DS, and DD in the other scenarios was not shown. This is mainly because the expected contribution of an average parent (*α*) and the regression coefficient of the contribution of an individual on its selective advantage (*β*) are inversely proportional to the number of parents as explained previously in equation (1). Consequently, the accounting percentage derived from SS, SD, DS, and DD for the rate of inbreeding (ΔF), (that is, the relative magnitude of ΔF in SS, SD, DS, and DD), resulted in almost the same for all three scenarios compared in the numbers of parents in SS, SD, DS, and DD, even if the absolute magnitude of ΔF derived from each of the four selection paths differed in the number of parents in each of the four selection paths.

### Correction derived from deviation from Poisson variance

Corrections for deviations in the variance of the family size from independent Poisson variances (×10^−4^) approximated by binomial distribution are shown in Table 6. The magnitude approximated by binomial distribution under the assumed selection percentages in the SS, SD, DS, and DD selection paths of 5%-12.5%–1%-70% and 1%-5%–1%-70% varied from −0.29×10^−4^ to −0.88×10^−4^, and −0.04×10^−4^ to −0.12×10^−4^, respectively. In comparison, the rates of inbreeding with Poisson family size without correction shown in Table 2 varied from 0.2×10^−2^ to 0.7×10^−2^. Therefore, because the magnitude of correction was much smaller than that of the rates of inbreeding with Poisson family size without correction, correction is unnecessary; thus the rates of inbreeding without correction (Table 2) are reasonable rates of inbreeding. However, the method in terms of the factorial moments [10] should be examined to confirm that the magnitude of correction is much smaller than those of ΔF with Poisson family size without correction.

Selection intensities and variance reduction coefficients should be adjusted by using the procedure from Wray and Thompson [4] in situations of few families with numerous candidates per family, for example, when the number of selected parents is only 5 or 10 [6]. Because we set the number of parents in SS at 20, 40, and 60, we did not adjust the selection intensity in the SS path. In addition, selection intensity in DD generally is much smaller than those in SS, SD, and DD selection paths. Consequently, when selection in DD is not performed, the selection intensity and reduction factor of the variance need to be set at zero in the DD selection path in the formula developed in the current study.

## CONCLUSION

We here developed a formula for calculating the rates of inbreeding in populations under selection based on GEBV. The population is selected along the four selection paths of SS (sires to breed sons), SD (sires to breed daughters), DS (dams to breed sons), and DD (dams to breed daughters). Assuming that the number and selection intensity of parents remained the same over the period of usage (several years) enabled us to regard generations as discrete generations. The effect on decreasing the rate of inbreeding was greater when the number of parents was increased than when the selection intensity was decreased, and both number of parents and the selection intensity in four-path selection emerged as major factors affecting the rate of inbreeding. In general, both genetic gain and ΔF tended to increase in line with any increase in selection intensity. Therefore, increasing the number of parents is one option for offsetting the increase in ΔF due to an increase in selection intensity. Especially, increasing the number of male parents would be effective, since the accounting percentage for the increase in ΔF from male parents is greater than that from female parents. When applied without correction for deviation of family size from Poisson distributions, the formula we developed here would be highly useful as a practical method for predicting the approximate rate of inbreeding (ΔF) in populations where selection is performed according to four-path programs.

## Figures and Tables

**Table 1:** Genetic variances and reliabilities of genomically enhanced breeding values in generation 0 and at the asymptote

Items	Genetic variances			Reliabilities		
	
	Single- trait (*h*^2^ = 0.3)	Single- trait (*h*^2^ = 0.1)	Index selection 1:1^1)^	Single- trait (*h*^2^ = 0.3)	Single- trait (*h*^2^ = 0.1)	Index selection 1:1
Generation 0	0.300	0.100	0.5386	0.5721	0.4836	0.5386
Male population						
1%-5%–1%-70%^2)^	0.2190	0.0771	0.4020	0.4138	0.3304	0.3846
5%-12.5%–1%-70%	0.2200	0.0774	0.4040	0.4164	0.3329	0.3861
Female population						
1%-5%–1%-70%	0.2365	0.0802	0.4202	0.4571	0.3563	0.3998
5%-12.5%–1%-70%	0.2367	0.0806	0.4225	0.4577	0.3590	0.4015

1) Economic weight is 1:1, genetic correlation of two traits (*h*^2^ = 0.3 and *h*^2^ = 0.1) is 0.4.

2) Selection percentage in selection paths of sires to breed sons (SS), sires to breed daughters (SD), dams to breed sons (DS), and dams to breed daughters (DD), respectively.

**Table 2:** Rate of inbreeding

Items	ΔF under selection of 5%-12.5%–1%-70%1)	ΔF under selection of 1%-5%–1%-70%	Ratio of ΔF ignoring selection to that including selection		Ratio of ΔF at 5%-12.5%–1%-70% to that at 1%-5%–1%-70%

			5%-12.5%–1%-70%	1%-5%–1%-70%	
20-50–100-7,000^2)^					
0^3)^	0.00376	0.00376			
1	0.00613	0.00701	0.613	0.558	0.875
2	0.00609	0.00698	0.613	0.558	0.873
3	0.00613	0.00702	0.614	0.559	0.873
40-100–200-14,000					
0	0.00188	0.00188			
1	0.00309	0.00348	0.609	0.540	0.887
2	0.00307	0.00346	0.609	0.540	0.888
3	0.00309	0.00349	0.609	0.540	0.886
60-150–300-21,000					
0	0.00125	0.00125			
1	0.00206	0.00235	0.608	0.534	0.879
2	0.00205	0.00233	0.608	0.534	0.879
3	0.00206	0.00235	0.608	0.534	0.878

SS, sires to breed sons; SD, sires to breed daughters; DS, dams to breed sons; DD, dams to breed daughters.

1) Selection percentages in SS, SD, DS, and DD, respectively.

2) Numbers of parents in SS, SD, DS, and DD, respectively.

3) 0, 1, 2, and 3 refer to rates of inbreeding from Gowe et al [8], which does not account for selection; single-trait selection (*h*^2^ = 0.3); single-trait selection (*h*^2^ = 0.1); and index selection based on two traits (*h*^2^ = 0.3 and *h*^2^ = 0.1) with equal economic weights, respectively.

**Table 3:** Effective population size (*N*_E_)

Items	*N*_E_ under selection of 5%-12.5%–1%-70%1)	*N*_E_ under selection of 1%-5%–1%-70%	Ratio of *N*_E_ at 5%-12.5%–1%-70% to that at 1%-5%–1%-70%
20-50–100-7,000^2)^			
0^3)^	132.9	132.9	1.000
1	81.5	74.2	1.099
2	82.1	74.9	1.096
3	81.6	74.2	1.100
40-100–200-14,000			
0	265.7	265.7	1.000
1	161.8	143.5	1.127
2	162.8	144.6	1.126
3	161.9	143.5	1.128
60-150–300-21,000			
0	398.6	398.6	1.000
1	242.2	212.8	1.138
2	243.6	214.1	1.138
3	242.3	212.7	1.139

SS, sires to breed sons; SD, sires to breed daughters; DS, dams to breed sons; DD, dams to breed daughters.

1) Selection percentages in SS, SD, DS, and DD, respectively.

2) Numbers of parents in SS, SD, DS, and DD, respectively.

3) 0, 1, 2, and 3 refer to rates of inbreeding from Gowe et al [8], which does not account for selection; single-trait selection (*h*^2^ = 0.3); single-trait selection (*h*^2^ = 0.1); and index selection based on two traits (*h*^2^ = 0.3 and *h*^2^ = 0.1) with equal economic weights, respectively.

**Table 4:** The expectation of the square of long-term contribution of individual in SS, SD, DS, and DD (×10^−4^)

Items	5%-12.5%–1%-70%^1)^				1%-5%–1%-70%^1)^			
	
	SS	SD	DS	DD	SS	SD	DS	DD
20-50–100-7,000^2)^								
1^3)^	2.4227	0.3532	0.1192	0.0000	2.8171	0.3593	0.1461	0.0000
2	2.4122	0.3532	0.1186	0.0000	2.8034	0.3614	0.1450	0.0000
3	2.4249	0.3531	0.1197	0.0000	2.8226	0.3605	0.1468	0.0000
40-100–200-14,000								
1	0.6114	0.0888	0.0304	0.0000	0.7058	0.0900	0.0369	0.0000
2	0.6086	0.0886	0.0302	0.0000	0.7013	0.0904	0.0366	0.0000
3	0.6119	0.0885	0.0305	0.0000	0.7068	0.0902	0.0371	0.0000
60-150–300-21,000								
1	0.2726	0.0395	0.0135	0.0000	0.3165	0.0401	0.0165	0.0000
2	0.2713	0.0394	0.0134	0.0000	0.3147	0.0403	0.0164	0.0000
3	0.2728	0.0394	0.0136	0.0000	0.3171	0.0402	0.0166	0.0000

SS, sires to breed sons; SD, sires to breed daughters; DS, dams to breed sons; DD, dams to breed daughters.

1) Selection percentage in SS, SD, DS, and DD.

2) Numbers of parents of SS, SD, DS, and DD.

3) 1, 2, and 3 refer to rates of inbreeding from single-trait selection (*h*^2^ = 0.3); single-trait selection (*h*^2^ = 0.1); and index selection based on two traits (*h*^2^ = 0.3 and *h*^2^ = 0.1) with equal economic weights, respectively.

**Table 5:** The accounting percentage derived from SS, SD, DS, and DD for the rate of inbreeding (ΔF) when the numbers of parents in SS, SD, DS, and DD are 40-100–200-14,000

Items	SS	SD	DS	DD
5%-12.5%–1%-70%^1)^				
1^2)^	0.574	0.322	0.101	0.004
2	0.574	0.322	0.101	0.004
3	0.574	0.321	0.101	0.004
1%-5%–1%-70%^1)^				
1	0.579	0.310	0.108	0.003
2	0.579	0.311	0.107	0.003
3	0.579	0.310	0.108	0.003

SS, sires to breed sons; SD, sires to breed daughters; DS, dams to breed sons; DD, dams to breed daughters.

1) Selection percentage in SS, SD, DS, and DD.

2) 1, 2, and 3 refer to rates of inbreeding from single-trait selection (*h*^2^ = 0.3); single-trait selection (*h*^2^ = 0.1); and index selection based on two traits (*h*^2^ = 0.3 and *h*^2^ = 0.1) with equal economic weights, respectively.

**Table 6:** Correction factors for deviations of the variance of the family size from independent Poisson variances (×10^−4^) approximated by binomial distribution

Items	Selection of 5%-12.5%–1%-70%^1)^	Selection of 1%-5%–1%-70%
20-50–100-7,000^2)^		
1^3)^	−0.870	−0.118
2	−0.883	−0.121
3	−0.875	−0.119
40-100–200-14,000		
1	−0.436	−0.059
2	−0.443	−0.060
3	−0.439	−0.060
60-150–300-21,000		
1	−0.291	−0.040
2	−0.295	−0.040
3	−0.293	−0.040

SS, sires to breed sons; SD, sires to breed daughters; DS, dams to breed sons; DD, dams to breed daughters.

1) Selection percentages in SS, SD, DS, and DD, respectively.

2) Numbers of parents in SS, SD, DS, and DD, respectively.

3) 1, 2, and 3 refer to rates of single trait selection (*h*^2^ = 0.3), single trait selection (*h*^2^ = 0.1), and index selection based on two traits (*h*^2^ = 0.3 and *h*^2^ = 0.1) with equal economic weights.

## SUPPLEMENTARY MATERIAL

Appendice A. Elements in matrix ***Π*** Appendice B. Elements in matrix and **λ** Appendice C. Elements of **Δ*****V****_SS,D,DS,or DD_*
