# Supplementary material for: Predicting the rate of inbreeding in populations undergoing four-path selection on genomically enhanced breeding values
Source: Anim Biosci. 2022 Jan 3;35(6):804–13. doi: 10.5713/ab.21.0350 (PMC9066037; doi:10.5713/ab.21.0350)
Supplement: Supplementary file 1 [file ab-21-0350-suppl.pdf]

Appendix A: Elements in matrix $\mathbf{\Pi}$ calculated according to [9] and [12].

Note that $\pi_{o,p} = [\text{cov}(s_p, s_o) - k_o \text{cov}(s_p, \hat{A}_o) cov(s_o, \hat{A}_o)/\sigma^2_{\hat{A}_o}]/\text{Var}(s_p)$, where subscripts p and o denote parent and offspring, with (co)variances taken before selection of the offspring; k_o is the reduction factor of the variance of the offspring due to selection of the offspring; and $\hat{A}_o$ is the GEBV of the offspring. For example,

$\pi_{SD,SS} = [\text{cov}(s_{p,SS}, s_{o,SD}) - k_{o,SD} \text{cov}(s_{p,SS}, \hat{A}_{o,SD}) cov(s_{o,SD}, \hat{A}_{o,SD})/\sigma^2_{\hat{A}_{o,SD}}]/\ \sigma^2_{s_{p,SS}}$,

$\text{cov}(s_{p,SS}, s_{o,SD}) = \text{cov}(s_{p,SS}, \frac{1}{2}(s_{p,SS} + s_{p,DS})) = \frac{1}{2}\sigma^2_{s_{p,SS}}$, and

$\text{cov}(s_{p,SS}, \hat{A}_{o,SD}) = \text{cov}(s_{p,SS}, r^2_{\hat{A}_{o,SD}} \text{A}_{\text{o,SD}}) = \text{cov}\left(s_{p,SS}, r^2_{\hat{A}_{o,SD}} \frac{1}{2}(s_{p,SS} + s_{p,DS})\right) = \frac{r^2_{\hat{A}_{o,SD}}}{2}\sigma^2_{s_{p,SS}}$, $cov(s_{o,SD}, \hat{A}_{o,SD}) = \sigma^2_{\hat{A}_{o,SD}}$.

Therefore, $\pi_{SD,SS} = \frac{1}{2}(1 - k_{o,SD} r^2_{\hat{A}_{o,SD}})$.

In the same way as for $\pi_{SD,SS}$,

$\pi_{SS,SS} = \frac{1}{2}\left(1 - k_{o,SS} r^2_{\hat{A}_{o,SS}}\right)$, $\pi_{SS,DS} = \frac{1}{2}\left(1 - k_{o,SS} r^2_{\hat{A}_{o,SS}}\right)$,

$\pi_{SD,DS} = \frac{1}{2}\left(1 - k_{o,SD} r^2_{\hat{A}_{o,SD}}\right)$, $\pi_{DS,SD} = \frac{1}{2}\left(1 - k_{o,DS} r^2_{\hat{A}_{o,DS}}\right)$,

$\pi_{DD,SD} = \frac{1}{2}\left(1 - k_{o,DD} r^2_{\hat{A}_{o,DD}}\right)$, $\pi_{DS,DD} = \frac{1}{2}\left(1 - k_{o,DS} r^2_{\hat{A}_{o,DS}}\right)$, and

$\pi_{DD,DD} = \frac{1}{2}\left(1 - k_{o,DD} r^2_{\hat{A}_{o,DD}}\right)$.

The other elements of $\mathbf{\Pi}$ are zero.

Appendix B: Elements in matrix and $\mathbf{\Lambda}$ calculated according to [9] and [12].

Note that $\lambda_{\text{o,p}} = p_o^{-1} b_{S,\hat{A}_O} b_{\hat{A}_O S_p}$, where $b_{S,\hat{\text{A}}_\text{O}} = p_o i_o \sigma^{-1}_{\hat{A}_o}$, and $b_{\hat{A}_o S_p} = cov(\hat{\text{A}}_\text{o}, S_p)/\sigma^2_{S_p}$,

where $\hat{\text{A}}_\text{O}$ is GEBV of the offspring, p_o is selected proportion of offspring,

$b_{S,\hat{A}_O}$ is the regression of the selection score (selected or not selected, i.e., S=1 or 0) of the offspring on its GEBV, i_o is selection intensity of the offspring selected, $\sigma^2_{\hat{A}_o}$ is the variance of the GEBV of the offspring, S_p is the selective advantage of the parent, and $b_{\hat{A}_o S_p}$ is the regression of the GEBV of the offspring on the selective advantage of the parent.

For example,

$b_{S,\hat{A}_{o,SD}}$ = $p_{o,SD} i_{o,SD} \sigma^{-1}_{\hat{A}_{o,SD}}$, $b_{\hat{A}_{o,SD} S_{p,SS}} = cov(\widehat{\mathrm{A}}_{\mathrm{o,SD}}, S_{p,SS})/\sigma^2_{S_{p,SS}}$,

$cov(\widehat{\mathrm{A}}_{\mathrm{o,SD}}, S_{p,SS})$ = $\mathrm{cov}(r^2_{\hat{A}_{o,SD}} \frac{1}{2}(\mathrm{s}_{p,SS} + \mathrm{s}_{p,DS}), S_{p,SS}) = \frac{r^2_{\hat{A}_{o,SD}}}{2} \sigma^2_{S_{p,SS}}$,

Therefore

$\lambda_{\mathrm{SD,SS}}$ = $i_{o,SD} \sigma^{-1}_{\hat{A}_{o,SD}} \frac{r^2_{\hat{A}_{o,SD}}}{2} = \frac{1}{2} i_{o,SD} \sigma^{-1}_{\hat{A}_{o,SD}} r^2_{\hat{A}_{o,SD}}$.

In the same way as for $\lambda_{\mathrm{SD,SS}}$,

$\lambda_{\mathrm{SS,SS}} = \frac{1}{2} i_{o,SS} \sigma^{-1}_{\hat{A}_{o,SS}} r^2_{\hat{A}_{o,SS}}$, $\lambda_{\mathrm{DS,SD}} = \frac{1}{2} i_{o,DS} \sigma^{-1}_{\hat{A}_{o,DS}} r^2_{\hat{A}_{o,DS}}$,

$\lambda_{\mathrm{DD,SD}} = \frac{1}{2} i_{o,DD} \sigma^{-1}_{\hat{A}_{o,DD}} r^2_{\hat{A}_{o,DD}}$, $\lambda_{\mathrm{SS,DS}} = \frac{1}{2} i_{o,SS} \sigma^{-1}_{\hat{A}_{o,SS}} r^2_{\hat{A}_{o,SS}}$,

$\lambda_{\mathrm{SD,DS}} = \frac{1}{2} i_{o,SD} \sigma^{-1}_{\hat{A}_{o,SD}} r^2_{\hat{A}_{o,SD}}$, $\lambda_{\mathrm{DS,DD}} = \frac{1}{2} i_{o,DS} \sigma^{-1}_{\hat{A}_{o,DS}} r^2_{\hat{A}_{o,DS}}$, and

$\lambda_{\mathrm{DD,DD}} = \frac{1}{2} i_{o,DD} \sigma^{-1}_{\hat{A}_{o,DD}} r^2_{\hat{A}_{o,DD}}$.

The other elements of $\mathbf{\Lambda}$ are zero.

Appendix C: Elements of $\Delta \boldsymbol{V}_{\boldsymbol{SS,SD,DS},or\ \boldsymbol{DD}}$

With a binomial distribution of family size, the deviation from a Poisson variance equals $np(1-p)-np=-np^2$, where n is the number of candidates and p is the selected proportion. Therefore,

$\Delta V_{SS,1,1} = -(\mathrm{N}_{DS}/N_{SS}) \times fmds \times p(1) \times p(1)$ and

$\Delta V_{SS,2,2} = -\left(\frac{\mathrm{N}_{DS}}{N_{SS}}\right) \times fmds \times p(2) \times p(2),$

where $\Delta V_{SS,i,j}$ is the (i, j) element in ΔV_{SS},

$fmds$ is the number of male offspring produced from a dam of DS, and

p(1) and *p(2)* are the selected proportions of SS and SD, respectively. The other elements of $\Delta \boldsymbol{V}_{\boldsymbol{SS}}$ are 0. Similarly,

$\Delta V_{SD,3,3} = -\left(\frac{\mathrm{N}_{DD}}{N_{SD}}\right) \times ffdd \times p(3) \times p(3)$ and

$\Delta V_{SD,4,4} = -\left(\frac{\mathrm{N}_{DD}}{N_{SD}}\right) \times ffdd \times p(3) \times p(3),$

where *p(3)* and *p(4)* are the selected proportions of DS and DD, respectively. The other elements of $\Delta \boldsymbol{V}_{\boldsymbol{SD}}$ are 0. Similarly,

$\Delta V_{DS,1,1} = -ffds \times p(1) \times p(1),$ $\Delta V_{DS,2,2} = -ffds \times p(2) \times p(2),$

$\Delta V_{DD,3,3} = -ffdd \times p(3) \times p(3),$ and $\Delta V_{DD,4,4} = -ffdd \times p(4) \times p(4).$

The other elements of $\Delta \boldsymbol{V}_{\boldsymbol{DS}}$ and $\Delta \boldsymbol{V}_{\boldsymbol{DD}}$ are 0.
